# Monitoring Pharmacologically Induced Immunosuppression by Immune Repertoire Sequencing to Detect Acute Allograft Rejection in Heart Transplant Patients: A Proof-of-Concept Diagnostic Accuracy Study

**DOI:** 10.1371/journal.pmed.1001890

**Published:** 2015-10-14

**Authors:** Christopher Vollmers, Iwijn De Vlaminck, Hannah A. Valantine, Lolita Penland, Helen Luikart, Calvin Strehl, Garrett Cohen, Kiran K. Khush, Stephen R. Quake

**Affiliations:** 1 Department of Bioengineering, Stanford University, Stanford, California, United States of America; 2 Department of Applied Physics, Stanford University, Stanford, California, United States of America; 3 Howard Hughes Medical Institute, Stanford, California, United States of America; 4 Division of Cardiovascular Medicine, Stanford University School of Medicine, Stanford, California, United States of America; Harvard University School of Medicine, UNITED STATES OF AMERICA

## Abstract

**Background:**

It remains difficult to predict and to measure the efficacy of pharmacological immunosuppression. We hypothesized that measuring the B-cell repertoire would enable assessment of the overall level of immunosuppression after heart transplantation.

**Methods and Findings:**

In this proof-of-concept study, we implemented a molecular-barcode-based immune repertoire sequencing assay that sensitively and accurately measures the isotype and clonal composition of the circulating B cell repertoire. We used this assay to measure the temporal response of the B cell repertoire to immunosuppression after heart transplantation. We selected a subset of 12 participants from a larger prospective cohort study (ClinicalTrials.gov NCT01985412) that is ongoing at Stanford Medical Center and for which enrollment started in March 2010. This subset of 12 participants was selected to represent post-heart-transplant events, with and without acute rejection (six participants with moderate-to-severe rejection and six without). We analyzed 130 samples from these patients, with an average follow-up period of 15 mo. Immune repertoire sequencing enables the measurement of a patient’s net state of immunosuppression (correlation with tacrolimus level, *r* = −0.867, 95% CI −0.968 to −0.523, *p* = 0.0014), as well as the diagnosis of acute allograft rejection, which is preceded by increased immune activity with a sensitivity of 71.4% (95% CI 30.3% to 94.9%) and a specificity of 82.0% (95% CI 72.1% to 89.1%) (cell-free donor-derived DNA as noninvasive gold standard). To illustrate the potential of immune repertoire sequencing to monitor atypical post-transplant trajectories, we analyzed two more patients, one with chronic infections and one with amyloidosis. A larger, prospective study will be needed to validate the power of immune repertoire sequencing to predict rejection events, as this proof-of-concept study is limited to a small number of patients who were selected based on several criteria including the availability of a large number of samples and the absence or presence of rejection events.

**Conclusions:**

If confirmed in larger, prospective studies, the method described here has potential applications in the tailored management of post-transplant immunosuppression and, more broadly, as a method for assessing the overall activity of the immune system.

## Abstract 2

A novel, proof-of-concept, diagnostic test for allograft rejection. The authors use a barcode-sequencing approach to determine the isotypes and clonal composition of circulating B cells in heart transplant patients.

## Editors' Summary

**Background:**

Fifty years ago, little could be done to help a person whose heart, liver, or kidneys had stopped working because of infection, injury, or a chronic disease process. Nowadays, solid organ transplantation gives more than 100,000 people worldwide a new lease on life every year. Many different organs can be successfully moved from one individual (the organ donor), who may be dead or alive, into another individual (the organ recipient). However, organ transplantation remains a complex and risky procedure. The organ recipient may not survive transplantation surgery, and sometimes the transplanted organ fails to work in its new owner. Another major risk for patients who receive an allograft (an organ from another person) is acute rejection. Our immune system protects us from pathogens (disease-causing organisms) by recognizing specific molecules (antigens) on an invader’s surface as foreign and initiating a sequence of events that kills the invader. Unfortunately, the immune system sometimes recognizes organ transplants as foreign and triggers acute transplant rejection. The chances of rejection can be minimized by “matching” the antigens on the donated organ to those on recipient’s organs and by giving the recipient immunosuppressive drugs such as prednisone and tacrolimus.

**Why Was This Study Done?:**

The primary aim of post-transplantation immunosuppression is to prevent acute rejection, which is associated with the activation of T cells (a type of lymphocyte or white blood cell). But immunosuppressive therapy also affects other immune system cells, including B cells, another type of lymphocyte. B cells make antibodies, molecules that recognize foreign antigens and tag pathogens for destruction by the immune system. It is important, therefore, to get the level of immunosuppression just right for every patient—too little and the transplanted organ will be rejected, too much and the patient will be susceptible to infections. In this proof-of-concept study, the researchers ask whether measurement of the B cell repertoire (the mixture of antibodies produced by a person’s B cells) can be used to assess the overall level of immunosuppression in patients after heart transplantation. When the body is threatened by a pathogen, naïve B cells undergo molecular processes called hypermutation and class-switch recombination. Hypermutation allows the immune system to adapt to new threats by changing the specificity and strength of antibody binding to antigens and involves changes in the “variable” regions of antibodies. Class-switch recombination changes the “isotype” of the antibody produced by a B cell by switching the constant region of the antibody’s “heavy chain.” Thus, determination of the composition of immunoglobulin heavy chain transcripts (nucleotide sequences that are translated into antibodies) in the blood could indicate the level of immunosuppression in an individual.

**What Did the Researchers Do and Find?:**

The researchers obtained blood samples collected over several months from twelve patients who had had a heart transplant, including six who had experienced a rejection event. Using a previously developed barcode-based immune repertoire sequencing assay, the researchers measured changes over time in the sequence and abundance of immunoglobulin heavy chains in the patients. They found that the fraction of antibody sequences related to activated B cells was inversely related to the level of tacrolimus in the patients’ blood and that antibody repertoire sequencing could detect immune activation during acute rejection events diagnosed by examining small pieces of the donated heart or by looking for donor-derived DNA in the recipients’ blood. Specifically, the immune repertoire sequencing assay diagnosed acute allograft rejection with a sensitivity of 71.4% and a specificity of 82.0%. That is, the assay correctly identified 71.4% of patients who had had a rejection event and 82.0% patients who had not had a rejection event.

**What Do These Findings Mean?:**

These findings suggest that measurement of the immune repertoire using a high-throughput sequencing assay might provide an accurate and sensitive way to manage immunosuppression in patients who have received an organ or tissue transplant. Importantly, however, these findings provide only proof of concept. Large prospective studies are now needed to confirm the ability of immune repertoire sequencing to predict rejection events and to compare this approach to existing assays for transplant rejection. If validated, immune repertoire sequencing may eventually make it possible to personalize immunosuppressive therapy after transplantation to ensure adequate suppression of the immune system’s response to the allograft while avoiding the many long-term consequences of over-immunosuppression.

**Additional Information:**

This list of resources contains links that can be accessed when viewing the PDF on a device or via the online version of the article at http://dx.doi.org/10.1371/journal.pmed.1001890.

- The UK National Health Service provides information about heart transplants, including a personal story
- The British Heart Foundation, a not-for-profit organization, also provides information about heart transplants
- The US National Heart, Lung, and Blood Institute provides further information about heart transplantation
- The British Liver Trust, a not-for-profit organization, explains why immunosuppression is needed after organ transplantation and describes some of the possible side effects of immunosuppressive drugs
- Wikipedia has pages about B cells and about organ transplantation (note that Wikipedia is a free online encyclopedia that anyone can edit; available in several languages)
- The MedlinePlus Medical Encyclopedia has pages on heart transplantation and on transplant rejection (in English and Spanish); MedlinePlus also provides links to further information about heart transplantation and organ transplantation

## Introduction

An enduring challenge in immunology is the lack of quantitative measurements of immune strength. Current clinical practice relies on very crude estimates of the activity of the immune system, such as white blood cell counts. In view of the lack of more predictive assays, pharmacological immunosuppressive therapy, e.g., in the context of post-organ-transplant therapy, is guided mainly by dosage and measurement of the concentration of immunosuppressive drugs in blood. In adult transplant recipients, these immunosuppressive drugs typically include induction agents (e.g., lymphocyte-depleting antibodies, such as anti-thymocyte globulin) followed by maintenance with a combination of corticosteroids (prednisone), calcineurin inhibitors (tacrolimus and cyclosporine), and anti-proliferative agents (most commonly mycophenolate mofetil [MMF]). Immunosuppression is therefore achieved by combining several drugs with distinct mechanisms of action. Calcineurin inhibitors, for example, inhibit or deplete T helper and T killer cells, respectively, and consequently reduce T-helper-cell-dependent B cell activation [1,2]. While corticosteroids have a general immunosuppressive effect, MMF specifically inhibits T and B cell division. Consequently, while post-transplant immunosuppressive therapy is primarily aimed at preventing acute rejection events associated with T cell activation, it will also, directly and indirectly, affect the composition of circulating naïve and activated B cells [3,4]. While in some cases therapeutic drug levels (e.g., trough or C_0_ levels of tacrolimus) can be monitored, these levels are more reflective of toxicity than therapeutic efficacy. Further, this approach does not account for individual differences in the response to immunosuppressive drugs, and frequently gives rise to complications related to over- or under-immunosuppression.

**Fig 1:**
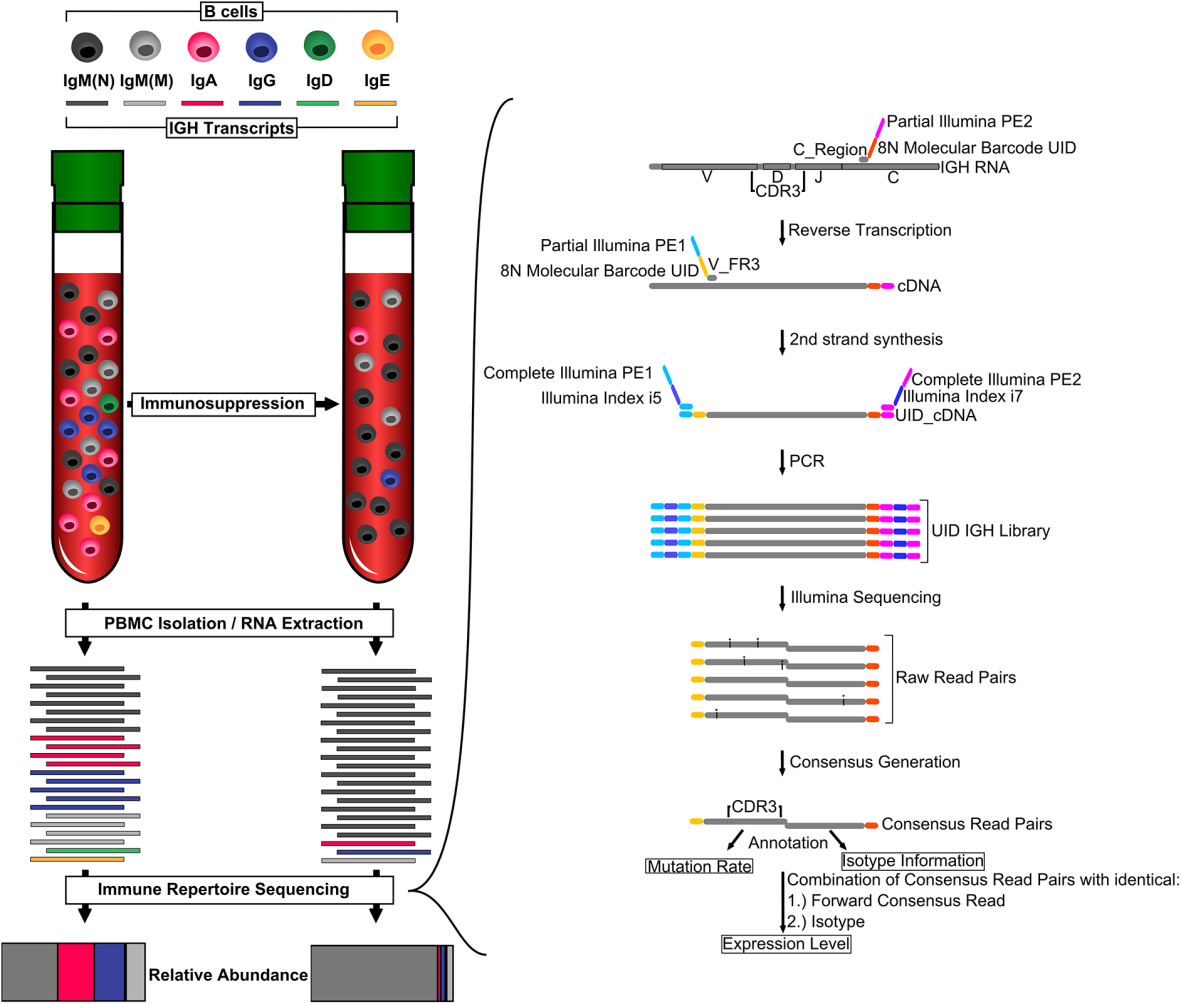
Monitoring overall immunosuppression. Organ transplant recipients receive immunosuppressive therapy, which usually includes calcineurin inhibitors. These drugs inhibit T cell activation, leading to fewer B cells expressing class-switched and mutated IGH transcripts. We measured overall immunosuppression using immune repertoire sequencing (IGH-Seq). A schematic of the IGH-Seq protocol and data analysis is shown on the right. C_Region, primer pool specific to constant region; CDR3, complementary determining region 3; PBMC, peripheral blood mononuclear cell; UID, unique identifier; V_FR3, primer pool specific to V segments.

Measuring the activity of the immune system directly would allow a more comprehensive understanding of the net state of the immune system. Here, we tested the hypothesis that immune repertoire sequencing of the B cell antibody heavy chain could provide an accurate and individualized measure of the activity of the adaptive immune response. The antibody heavy chain IGH transcript is unique as its expression changes fundamentally, not only in abundance but also in sequence, when a B cell is activated. B cells can undergo hypermutation and class-switch recombination when activated. Activated B cells express high levels of mutated antibodies of the IgG and IgA isotypes, while naïve B cells express non-mutated IgM antibodies at low levels (Fig 1). A lower abundance of class-switched sequences indicates a lower number of activated B cells and, consequently, lower activity of the adaptive immune system. Indeed, microarray studies have previously demonstrated that immunosuppressed individuals display reduced expression of antibody transcripts in general, and class-switched antibody transcripts in particular [5]. Furthermore, reduced hypermutation in IgM sequences following immunosuppressive therapy has previously been observed [6].

Immune repertoire sequencing relies on high-throughput sequencers to identify the composition of IGH transcripts in a blood sample, and has been used for various applications, including tracking of minimal residual disease in acute lymphoblastic leukemia, studying age-related changes in the immune system, and identifying the memory B cell response to influenza vaccination [7–11]. In a previous study, we described a molecular-barcoded immune repertoire sequencing protocol that is able to determine the sequence and abundance of IGH transcripts with unprecedented accuracy [7].

Here, we determined whether the immune repertoire sequencing approach enables quantification of loss of immune activity in response to pharmacological immunosuppression. We found that the fraction of antibody sequences related to activated B cells is inversely correlated with the dosage of immunosuppressive drugs, and could be used as a marker of net immunosuppression. We furthermore found that antibody repertoire sequencing detects immune activation during acute rejection events, as diagnosed by cell-free donor-derived DNA (cfdDNA) levels or endomyocardial biopsies [12,13].

## Methods

### Patient Selection

This work was approved by the Stanford University Institutional Review Board (protocol 17666), and all individuals provided written informed consent.

The specific goal of this proof-of-concept study was to test the clinical utility of immune repertoire sequencing to measure immunosuppression and predict acute rejection after heart transplantation. Participants included in this study were selected from a larger, ongoing prospective cohort study that involves recruitment of de novo pediatric and adult heart transplant candidates at Stanford Medical Center. The original cohort study was designed to determine the utility of cfdDNA for acute rejection surveillance after heart transplantation, and has been described previously [13]. Participant enrollment started in March 2010 and continues to this time, with patient follow-up for 5 y after transplantation. Multi-organ transplant candidates and recipients of repeat heart transplantation are excluded. Participants are recruited while on the waiting list for heart transplantation, and include patients requiring transplantation for a variety of end-stage heart diseases, including congenital heart disease, ischemic cardiomyopathy, dilated cardiomyopathy, valvular heart disease, and other etiologies.

We selected 12 adult transplant recipients from this larger prospective cohort study to represent patients with and without acute rejection events who were otherwise free of severe infections and other immune-related disorders. All buffy coat samples available at the onset of the study were analyzed, resulting in an average of 15 mo of follow-up for the 12 participants. Samples collected after this point were not included in the study. The main outcome measures of this study were the abundance and composition of the circulating B cell repertoire, in order to determine the temporal response of the B cell repertoire to immunosuppression after heart transplantation. We were interested in investigating the temporal dynamics of the B cell repertoire and therefore selected patients for whom a relatively large number of longitudinal samples were available. Among the patients analyzed in this study, six had experienced a rejection-free post-transplant course (patients 1–6; endomyocardial biopsy grade < 2R and cfdDNA below a previously determined threshold of 1% donor DNA), and six patients had experienced a moderate-to-severe rejection event as determined by endomyocardial biopsy grade ≥ 2R or elevated cfdDNA levels (patients 7–12) (S1 Table). We analyzed two additional time courses from patients with atypical post-transplant courses: patient 13 had experienced multiple opportunistic infections, and patient 14 had had a stem cell transplant for amyloid light chain (AL) amyloidosis. A total of 155 samples were analyzed (130 from patients 1–12 and 25 from patients 13 and 14; average of 11 samples per patient).

### Post-Transplant Therapeutic Protocol

Post-transplant immunosuppression consisted of methylprednisolone 500 mg administered immediately postoperatively, followed by 125 mg every 8 h for three doses. Anti-thymocyte globulin (rATG) 1 mg/kg was administered on postoperative days 1, 2, and 3. Maintenance immunosuppression consisted of prednisone 20 mg twice daily starting on postoperative day 1 and tapered to <0.1 mg/kg/d by the sixth postoperative month, and tapered further if endomyocardial biopsies showed no evidence of cellular rejection. Tacrolimus was started on postoperative day 1, and dosing was adjusted to maintain a level of 10–15 ng/ml during months 0–6, 7–10 ng/ml during months 6–12, and 5–10 ng/ml thereafter. MMF was started at 1,000 mg twice daily on postoperative day 1, and dose adjustments were made, if required, in response to leukopenia. All heart transplant recipients, except for those in whom both the donor and recipient had no history of prior cytomegalovirus infection (i.e., donor and recipient cytomegalovirus IgG-negative), received anti-viral prophylaxis with valganciclovir 900 mg twice daily for 2 wk, then 900 mg daily until 6 mo post-transplant, followed by 450 mg daily until 12 mo post-transplant, at which point anti-viral prophylaxis was discontinued. Valganciclovir dose reductions were made in the setting of leukopenia. Anti-fungal prophylaxis consisted of itraconazole 300 mg daily for the first 3 mo post-transplant, and prophylaxis against *Pneumocystis jiroveci* infection consisted of trimethoprim 80 mg/sulfamethoxazole daily. All heart transplant recipients were monitored for acute cellular rejection (ACR) by surveillance endomyocardial biopsies performed at scheduled intervals after transplant: weekly during the first month, biweekly until the third month, monthly until the sixth month, and then at months 9, 12, 16, 20, and 24. Biopsies were graded according to the International Society for Heart and Lung Transplantation 2004 revised grading scale (0, 1R, 2R, 3R). Blood was sampled at the same time points, and when blood sampling and endomyocardial biopsies were performed on the same day, care was taken to ensure that blood was collected prior to the biopsy procedure. A subset of heart transplant recipients also had blood samples collected on post-transplant days 1 and 7. There were no adverse effects of performing biopsies or blood draws.

### Whole Blood Processing and Nucleic Acid Extraction

Plasma and buffy coat samples were extracted from whole blood by sequential centrifugation within 3 h of sample collection and stored at −80°C. When required for analysis, plasma samples were thawed, and circulating DNA was immediately extracted from 0.5–1 ml of plasma using the QIAamp Circulating Nucleic Acid Kit (Qiagen). RNA was extracted from buffy coat samples using TRIzol Reagent (Life Technologies) followed by RNeasy column (Qiagen) extraction.

### cfdDNA Library Preparation

Libraries were generated by a single researcher who was blinded to all clinical data until after sequencing and initial data analyses had occurred. Sequencing libraries were prepared from the purified patient plasma DNA using the NEBNext DNA Library Prep Master Mix Set for Illumina with standard Illumina indexed adapters, or using a microfluidics-based automated library preparation platform (Mondrian SP Ovation SP Ultralow Library System, Nugen). Libraries were characterized using the Agilent 2100 Bioanalyzer (high-sensitivity DNA kit) and quantified by quantitative PCR.

### Immune Repertoire Library Preparation

Libraries were generated by two investigators (L. P. and C. V.) who were blinded to all clinical data until after sequencing and initial data analyses had occurred. 0.5–1 μm of total RNA were used as input for library preparation. Reverse transcription used SuperScript III Enzyme (Life Technologies) and primers for all five isotype constant regions containing eight random nucleotides and partial Illumina adapters. Second-strand synthesis was done using Phusion High-Fidelity DNA Polymerase (New England Biolabs) and primers for framing region 3 off all IGH variable (V) segments containing eight random nucleotides and partial Illumina adapters. The combined 16 random nucleotides (eight constant region primers and eight V segment primers) serve as a unique identifier (UID) for each IGH transcript molecule. Double-stranded cDNA was purified two times using AMPure XP beads at a ratio of 1:1. Double-stranded cDNA was amplified with Platinum Taq DNA Polymerase High Fidelity (Life Technologies) and two custom Illumina indexing primers completing the Illumina adapters and containing two Illumina index sequences (i5 and i7). Final sequencing libraries were generated by purifying the PCR product using AMPure XP beads at a ratio of 1:1 (Fig 1). Primer sequences were described previously [7].

### Sequencing and Data Analysis

Immune repertoire libraries were grouped into library pools with up to 50 libraries with compatible Illumina indices and sequenced on the Illumina HiSeq 2000 or MiSeq using 2 × 100 bp or 2 × 150 bp protocols. The sequencing runs yielded between 1 and 10 million raw reads per sample. Raw reads were split into UID groups with unique 16-nucleotide identifiers. Every UID group defined a single original IGH transcript molecule, and consensus reads were built for forward and reverse reads of each UID group, correcting sequencing errors for UID groups with coverage of two or more raw sequencing reads. While this approach does not result in the correction of sequencing errors for UID groups with a coverage of one, it still identifies these groups as originating from a unique molecule, thereby aiding in quantification. Forward consensus reads were annotated by alignments to V segments using BLAST, yielding mutation rate information. Reverse consensus reads were aligned to constant regions using BLAST [14], yielding isotype information. To compare samples, data for each sample were randomly subsampled to 4,000 aligned consensus read pairs. Samples producing fewer than 4,000 aligned consensus reads were discarded. UID groups with identical forward consensus reads (containing complementary determining region 3 sequence information) and isotype were assumed to originate from the same B cell clone and were combined to determine expression levels of each antibody IGH transcript. Quantification and visualization was done using custom scripts based on NumPy/SciPy/Matplotlib/Python [15–18] scripts, which are available in S1 Data and Scripts.

### Anellovirus Load

Shotgun DNA sequencing reads generated from the cfdDNA libraries that did not map to the human genome were aligned to a reference of viral, bacterial, and fungal genomes (BLAST). The relative genomic abundance of Anelloviridae virus was calculated with GRAMMy, a tool that utilizes BLAST-derived nucleic acid sequence similarity data to estimate the relative abundance of species [19].

### Statistical Analysis

**Fig 2:**
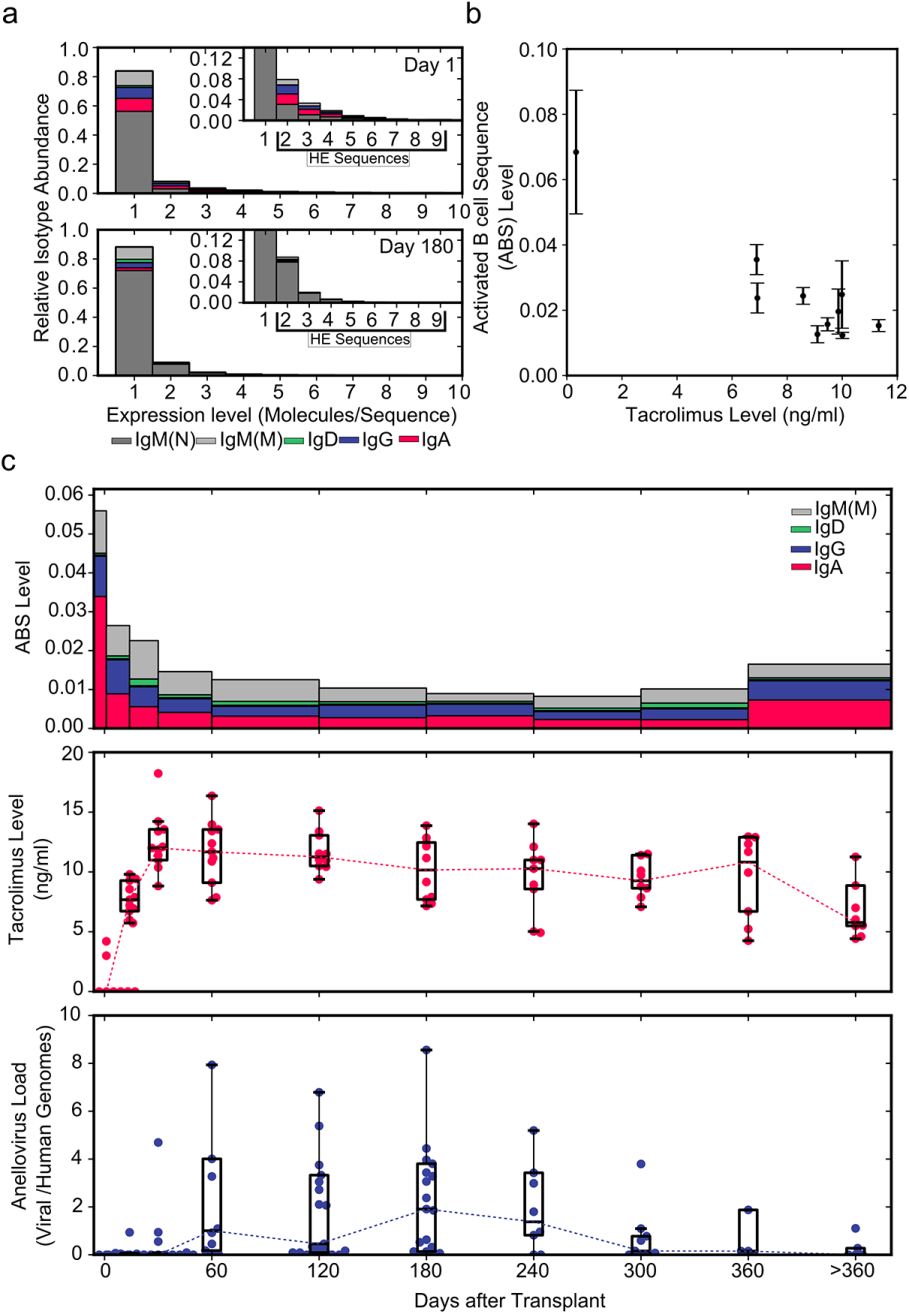
Using immune repertoire sequencing to measure immunosuppression. (A) Relative IGH sequence composition at increasing expression levels (molecules/sequence) is shown across all individuals (*n* = 12) in a histogram for days 1 and 180 post-transplant. Bar colors indicate the contribution of different immunoglobulin isotypes. Inserts show rescaled histograms. Highly expressed (HE) sequences (sequences represented by more than one molecule) are labeled in the insert. (B) ABS level (defined as the ratio of all highly expressed IgA, IgD, IgE, IgG, and mutated IgM sequences to the total number of molecules) as a function of tacrolimus trough level (*n* = 130) (average ± standard error of the mean). (C) Median ABS level (colors indicate isotype contribution), tacrolimus trough level, and total anellovirus load for all study patients (*n* = 12) during the first year after transplant.

Differences between groups (no evidence of rejection, mild acute rejection diagnosed by endomyocardial biopsy, moderate-to-severe acute rejection diagnosed by endomyocardial biopsy, and rejection diagnosed by elevated cfdDNA) were tested using Mann-Whitney U tests. C-statistic, sensitivity, and specificity were determined using the sklearn.metrics package for Python [20]. Confidence intervals for sensitivity, specificity, positive predictive value, and negative predictive value were calculated according to the efficient-score method described by Wilson and Newcombe [21,22]. Confidence intervals for the c-statistic were determined using bootstrapping. The confidence interval for the Pearson correlation (*r*) between activated B cell sequence (ABS) level (defined as the ratio of all highly expressed IgA, IgD, IgG, IgE, and mutated IgM sequences to the total number of molecules) and tacrolimus level (Fig 2B) was calculated based on the Fisher *r*-to-*z* transformation.

### Data Sharing

The shotgun DNA sequence data have been deposited in the Sequence Read Archive (SRP034946). The immune repertoire sequence data have been deposited in the Sequence Read Archive (PRJNA260905).

## Results

In this proof-of-concept study, we analyzed 130 blood samples from 12 patients who underwent pharmacological immunosuppression to prevent allograft rejection after heart transplantation. These individuals were a subset of a larger prospective cohort study [13] and were selected to represent patients with and without acute rejection events who were otherwise free of severe infections or other immune-related disorders. Starting on the first day post-transplant, until up to 2 y after transplantation, samples were collected at predetermined time points, with decreasing frequency as the study progressed. Concurrently with immune repertoire measurements (Fig 1), allograft health was monitored using cfdDNA levels and endomyocardial biopsies. We used DNA extracted from the plasma fraction of whole blood samples for the cfdDNA measurements and RNA extracted from the buffy coat for the immune repertoire sequencing protocol. Molecular barcodes were incorporated during reverse transcription of the extracted RNA and second-strand synthesis of the resulting cDNA. The barcoded double-stranded cDNA molecules were amplified as described previously (Fig 1) [7], and sequenced on HiSeq 2000 (2 × 100 bp) or MiSeq (2 × 150 bp) platforms. Expression level, mutation rate, and isotype were determined for each antibody IGH transcript (Fig 1).

### Immunosuppression Causes Shift in Isotype Expression

We first determined whether we could measure the effects of immunosuppression on the activity of the immune system using immune repertoire sequencing of the antibody IGH transcript. To this end, we pooled data for all participants in the study and separated antibody IGH transcript sequences by expression level.

Lowly expressed sequences were defined as antibody IGH transcript sequences that were represented by only one molecule and that were likely expressed by naïve or inactive B cells. Highly expressed sequences were defined as antibody IGH transcript sequences that were represented by more than one molecule and that were likely expressed by activated B cells such as plasmablasts [7,23].

We compared all data, including time points with diagnosed rejection events, pooled from the 12 participants of the main study at the beginning of (day 1) and 6 mo into (day 180, 20 d before first rejection event) immunosuppressive therapy. When normalized to the total number of molecules analyzed, the number of highly expressed sequences did not vary substantially between day 1 (2,131 highly expressed sequences/19,845 molecules; ratio 0.107) and day 180 (8,018 highly expressed sequences/79,852 molecules; ratio 0.100). Interestingly, a much larger change was observed in the composition of highly expressed sequences. Highly expressed IgA, IgD, IgE, IgG, and mutated IgM sequences decreased from 1,296/19,845 molecules (ratio 0.065) at day 1 to 847/79,852 molecules (ratio 0.011) by day 180 after the induction of immunosuppression (Fig 2A and 2C). Based on this change we defined the ABS level as this ratio of all highly expressed IgA, IgD, IgE, IgG, and mutated IgM sequences to the total number of molecules. The decrease in ABS level after the induction of immunosuppressive therapy indicated that it is possible to quantify the level of immunosuppression using immune repertoire sequencing.

We found that ABS level decreased rapidly in the first month, and the initial rapid decrease was followed by a slow decline over subsequent months. ABS level approached approximately 15% of the original level after 10 mo, after which the ABS level increased in response to tapering of immunosuppressive therapy. The decrease in ABS level was strongly and negatively correlated with blood tacrolimus concentration (Pearson *r* = −0.867, 95% CI −0.968 to −0.523, *p* = 0.0014; Fig 2B and 2C).

By reducing the activity of the immune system, immunosuppressive therapy also alters selective pressure on bacterial and viral populations in the human body. We previously showed that the composition of the viral population, as measured by sequencing of cell-free DNA in plasma, is significantly affected by post-transplant immunosuppressive therapy [12]. In particular, we found that the number of sequences related to the Anelloviridae family of viruses increased dramatically between months 1 and 10 after transplantation. The apparent inverse correlation of anellovirus load and ABS level observed in this work (Fig 2C) further confirmed our observation that ABS level reflects the overall level of immunosuppression.

### Elevated ABS Levels Indicate Acute Rejection Events

**Fig 3:**
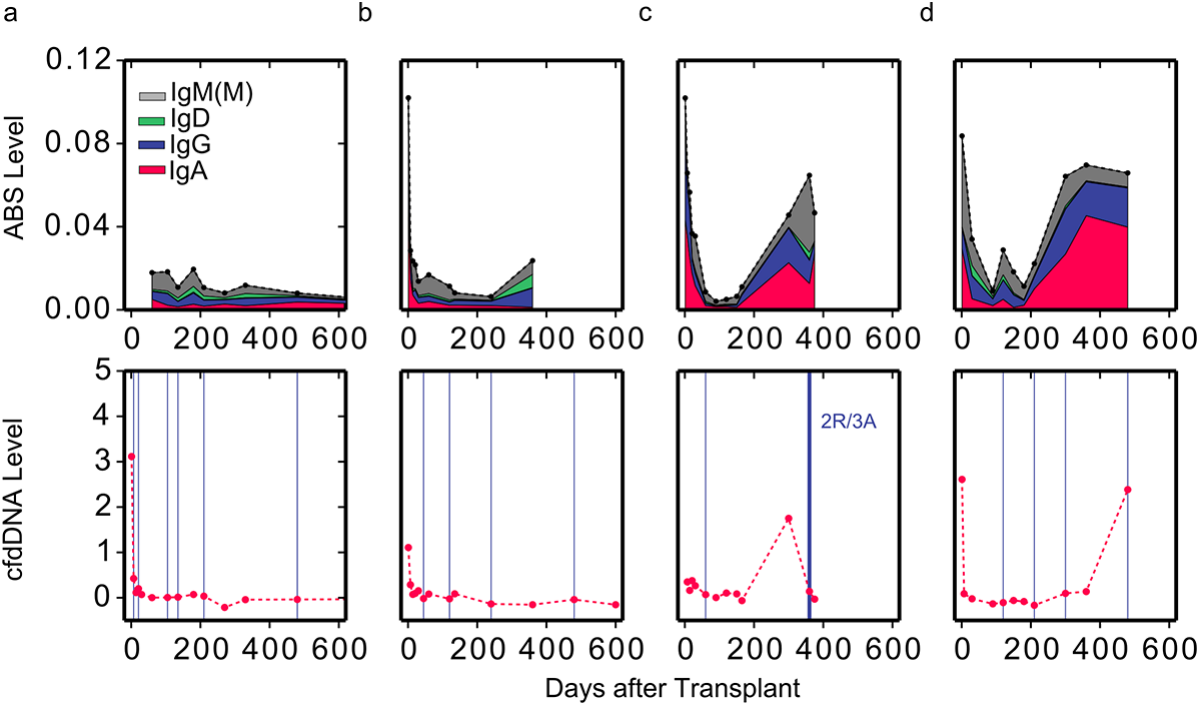
Reduced level of overall immunosuppression correlates with acute rejection events. ABS levels are shown for two individuals with (C and D) and two individuals without (A and B) moderate-to-severe acute rejection events (top). Colors indicate isotype contribution. cfdDNA levels are shown for the same individuals (bottom). Biopsy grades are shown as vertical blue lines (bottom). Thin lines indicate mild ACR events (grade 1R), and thick lines indicate moderate-to-severe ACR events (grade ≥ 2R).

Next, we examined whether ABS levels are also informative of acute rejection events. We examined ABS level in four patients, two of whom experienced acute rejection as diagnosed by either cfdDNA level (>1% cfdDNA) or endomyocardial biopsy. While the ABS level of individuals without rejection remained at baseline throughout the study period (Fig 3A and 3B), ABS level in individuals with ACR increased dramatically at the time of or prior to ACR, indicating increased activity of the alloimmune response. The ABS levels of individuals increased up to 8-fold (Fig 3C and 3D), with the median ABS level elevated approximately 3-fold. Interestingly, in one example, both cfdDNA and ABS levels were elevated, yet endomyocardial biopsy, graded by a pathologist, indicated no rejection event at this time point (Fig 3D). This discrepancy suggests that ABS level can be used as an additional independent marker of acute rejection when cfdDNA measurements disagree with biopsy results.

### ABS Levels Correlate Well with Endomyocardial Biopsy Results and cfdDNA Levels

**Fig 4:**
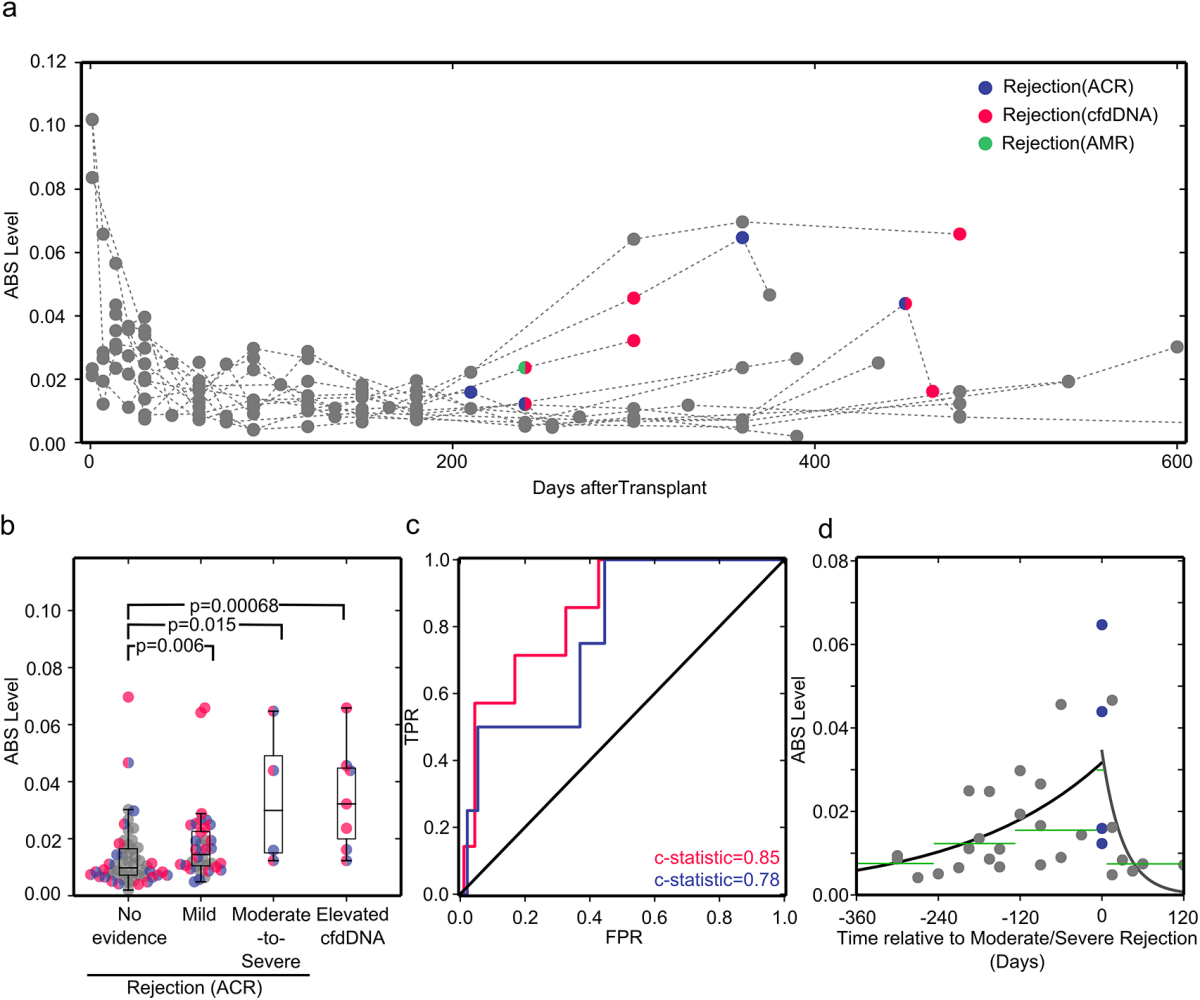
ABS levels correlate well with cfdDNA and endomyocardial biopsy results. (A) ABS levels plotted for all study participants. Acute rejection events as diagnosed by endomyocardial biopsy (ACR, violet; acute antibody-mediated rejection [AMR], green) or cfdDNA level (pink) are shown in color. (B) Samples are classified by rejection status diagnosed by biopsy as no evidence of rejection, mild ACR (biopsy grade 1R), or moderate-to-severe ACR (biopsy grade ≥ 2R), and by rejection status diagnosed by the cfdDNA assay. Samples are colored as in (A) if at any point the organ recipient experienced a serious rejection event. As biopsy grades are independent of cfdDNA measurements, there is overlap between samples in the elevated cfdDNA and biopsy-proven rejection bins. The groups were compared using a one-sided Mann-Whitney U test, which has high efficiency for both normally and non-normally distributed datasets. (C) Receiver operating characteristic (ROC) curves for ABS levels compared to cfdDNA (pink) and endomyocardial biopsy (violet) results. FPR, false rejection rate; TPR, true positive rate. (D) ABS levels measured before and after rejection diagnosis. The black line shows the single-exponent fit of ABS levels measured prior to rejection, *y* = *a* × (exp[*b* × *t*]), with best fit values (least squares) of *a* = 0.0317 and *b* = 0.00468. The gray line shows the single-exponent fit of ABS levels after diagnosis and during treatment of rejection: *y* = *a* × (exp[*b*× *t*]), with best fit values (least squares) of *a* = 0.0344 and *b* = −0.0324. Green lines show the median ABS levels in the covered time periods.

To investigate the performance of the immune repertoire sequencing assay compared to cfdDNA and biopsy measurements, we plotted the ABS levels of all 12 study patients, highlighting acute rejection events as diagnosed by cfdDNA and endomyocardial biopsy (Figs 4A and S1). The induction of immunosuppression reduced ABS level to a baseline level of around 0.02 after about 1 mo (Fig 4A). ABS levels subsequently measured during many acute rejection were elevated above this baseline (Fig 4A).

For a systematic analysis, we grouped samples according to biopsy and cfdDNA results. While all endomyocardial biopsies were tested for ACR, only some biopsies were also tested for antibody-mediated rejection, based on clinical parameters, thereby preventing systematic analysis for this type of acute rejection. In the cohort studied, antibody-mediated rejection was clinically reported at only one time point (Fig 4A). Therefore, we separated samples into no evidence of rejection (negative cfdDNA and biopsy grade 0), mild ACR (biopsy grade 1R), moderate-to-severe ACR (biopsy grade ≥ 2R), and rejection diagnosed by cfdDNA alone (“elevated cfdDNA”). We found that all subgroups with acute rejection had significantly different ABS levels compared to the non-rejection subgroup. The samples corresponding to acute rejection detected through cfdDNA measurement alone had the most significant *p*-value, followed by the samples corresponding to moderate-to-severe rejection (Fig 4B). We determined the area under the ROC curve for different ABS value thresholds, and found very good concordance between ABS levels and measurements of cfdDNA (c-statistic = 0.85 [95% CI 0.7 to 0.97], sensitivity 71.4% [95% CI 30.3% to 94.9%], specificity 82.0% [95% CI 72.1% to 89.1%], threshold 0.023) and between ABS levels and biopsy grades (c-statistic = 0.78 [95% CI 0.54 to 0.99]) (Fig 4C). When compared to cfdDNA as the noninvasive gold standard, the ABS assay achieved a positive predictive value of 0.24 (95% CI 0.09 to 0.48) and a negative predictive value of 0.97 (95% CI 0.9 to 1). The low positive predictive value can be partly explained by elevated ABS levels at time points preceding elevated cfdDNA levels. While these time points are categorized as false positives, they can provide valuable information about the patient’s immune activity prior to acute allograft rejection events.

To investigate the potential for early diagnosis, we analyzed the temporal dynamics of ABS level prior to a biopsy-proven moderate-to-severe rejection event (Fig 4D). A gradual and significant increase in ABS level is observed leading up to rejection episodes. Compared to the baseline ABS level (>8 mo before rejection), the median ABS level increased 2-fold within the 4-mo time window prior to rejection events (*p* = 0.021, Mann-Whitney U test). We furthermore investigated the response of the antibody repertoire to treatment of acute rejection (Fig 4D). Here, successful rejection treatment gave rise to a rapid decrease in ABS level, reaching a level comparable to the baseline level as early as 1 mo after initiation of treatment. Together, these data highlight the potential for early diagnosis and the use of these measurements to evaluate the efficacy of rejection therapy.

### ABS Level Correlates with Infections, Drug Therapy, and B Cell Disorders

**Fig 5:**
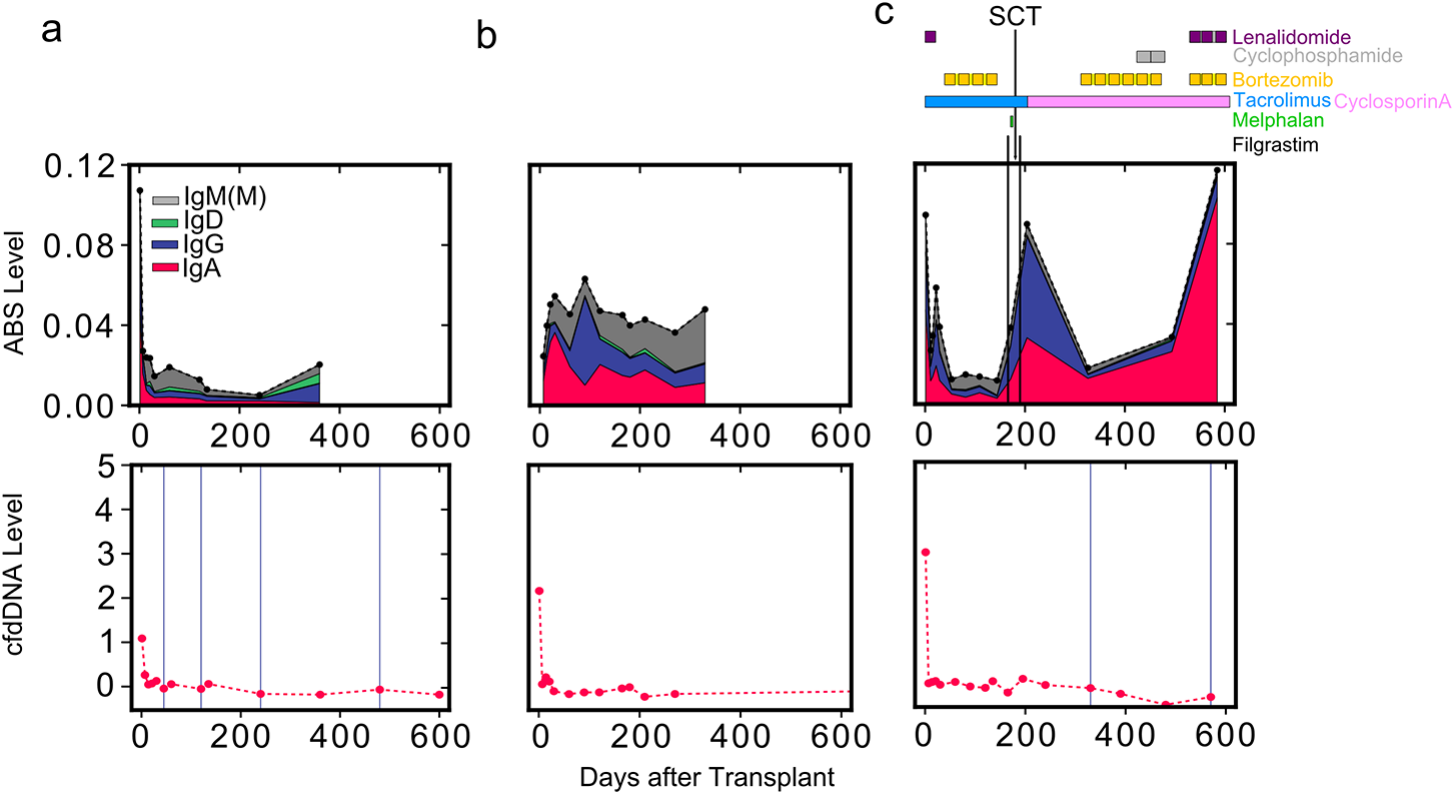
Reduced net immunosuppression correlates with acute rejection. ABS level is shown for three individuals: (A) a heart transplant recipient without moderate-to-severe rejection events, (B) a heart transplant recipient with infectious complications, and (C) a heart transplant recipient with AL amyloidosis. For the patient with AL amyloidosis (C), the approximate schedule of drug treatment is indicated at the top of the panel. Colors indicate isotype contribution. cfdDNA level is shown for the same individuals (bottom). Biopsy grades are shown as vertical blue lines (bottom): thin lines indicate mild rejection events (grade 1R), and thick lines indicate moderate-to-severe rejection events (grade ≥ 2R). SCT, stem cell transplant.

We next sought to examine the effect of opportunistic infections and B cell disorders on antibody repertoire sequencing measurements. To this end, we tracked ABS level in 25 samples from two additional heart transplant recipients. The first patient experienced several infections diagnosed both before and after transplantation, including cytomegalovirus, Epstein-Barr virus, hepatitis B virus, varicella zoster virus, staphylococcus, parainfluenza 3, and *Mycobacterium avium*. Interestingly, ABS level was elevated throughout the course of the study in this individual (Fig 5B) compared to a representative non-rejection individual free of infectious complications (Fig 5A). The second patient had AL amyloidosis, a B cell dyscrasia characterized by excessive antibody light chain production. End-stage cardiomyopathy related to AL amyloidosis was the indication for transplantation in this patient, who received several additional immunosuppressive and chemotherapeutic agents post-transplant in order to treat this disorder (Fig 5C). Two events over the course of his treatment highlight the unique aspects of this case: (1) this patient received an autologous stem cell transplant 6 mo after the heart transplant surgery, and (2) he was repeatedly treated with the drug filgrastim (granulocyte colony stimulating factor) to increase the number of hematopoietic stem cells and granulocytes in his blood prior to and after bone marrow transplantation. We detected a 6-fold increase in this patient’s ABS level following filgrastim administration (Fig 5C). The steep increase in ABS level that we observed is in agreement with a previously reported increase in the number of activated B cells following filgrastim treatment [24,25]. This patient continued to produce an excess of antibody light chains, and a more than 10-fold increase in ABS abundance was detected in the year following bone marrow transplantation, based entirely on an expansion of IgA sequences, despite treatment with immunosuppressive agents. While amyloidosis is generally considered to be caused by a single aberrant B cell clone, this measured increase in IgA was polyclonal (S2 Fig), suggesting a possible polyclonal component of the disorder. These two cases illustrate the potential of immune repertoire sequencing to monitor infectious complications, immunosuppressive drug treatment, and B cell disorders.

## Discussion

Heart transplantation is considered the best therapeutic option for the treatment of eligible patients with end-stage heart disease [26]. After transplantation, patient management focuses on the difficult task of achieving a balance between over- and under-immunosuppression. The immune repertoire sequencing assay presented here has the potential to measure the overall level of immunosuppression and to detect episodes of acute rejection after heart transplantation. Yet, further validation of the assay by a prospective study is needed, as this proof-of-concept study included a relatively small number of patients, and the patients included in this study were selected based on their rejection status and the availability of a high number of longitudinal samples.

Samples were collected longitudinally from the heart transplant recipients in this study, and immune repertoire measurements were compared to biopsy grades, tacrolimus levels, and the level of cfdDNA, as determined by shotgun sequencing. This longitudinal sampling enabled us to closely monitor the dynamics of immunosuppression. Measurements of cfdDNA and biopsies performed in parallel to the immune repertoire assay made it possible to compare our assay to both a noninvasive independent assay (cfdDNA) as well as the invasive diagnostic gold standard for acute organ rejection (endomyocardial biopsy). This approach provided us with an unprecedented view of the immune response to pharmacological immunosuppression, and how it escapes sufficient suppression, resulting in acute rejection.

Several commercially available assays aim to accomplish these goals [27,28] but have shown poor predictive accuracy [29,30]. The ImmuKnow (Cylex) assay measures the ability of T cells to be activated in vitro. The AlloMap assay (CareDx) uses microarray analysis to measure the expression levels of 11 genes that change in response to immunosuppression. It provides a numerical score that is affected by the composition and activation state of the various cell populations sampled. While this score is easy to interpret from a clinical perspective, the biological basis for the score is highly convoluted, and the score does not provide useful information outside of adult heart transplantation. In contrast, the immune repertoire sequencing assay that we developed queries the highly informative and well-understood IGH locus. After activation, B cells express IGH transcripts in a way that is both quantitatively (higher expression levels) and qualitatively (hypermutation and isotype class switch recombination) distinct from that of non-activated B cells. B cell activation requires activated T cells and takes several days to complete. Therefore, by quantifying the number of unique IGH sequences expressed by activated B cells (ABSs), our measurement integrates the level of immune activation over an extended period of time and has better test performance characteristics for detecting rejection events diagnosed by biopsy than the AlloMap assay (c-statistic ABS = 0.78 versus c-statistic AlloMap = 0.72; data from [31]).

The immune repertoire assay presented here is very efficient in terms of both sample preparation and sequencing: in this study we implemented a dual indexing strategy that allowed us to multiplex more than 200 samples on a single HiSeq 2000 flow-cell lane, thereby reducing the cost per sample—including sample preparation—to approximately US$40. The assay furthermore provides a value based on internal controls that does not rely on external normalization, and can be performed on small and low-quality samples. In addition to our main focus on immunosuppression and immune activation via rejection, we examined the dynamics of the B cell repertoire for two patients with unusual post-transplant courses: one patient with severe infectious complications and one patient with a B cell disorder. These case studies indicate the potential of immune repertoire sequencing in the management of a diverse set of post-transplant complications and also illustrate the potential value of combining immune repertoire sequencing with other assays, such as the measurement of cfdDNA in plasma, in order to discriminate between possible causes of immune activation.

Prospective studies are required to validate this assay in a larger patient population, to compare immune repertoire sequencing assay results to other noninvasive screening tests for heart transplant complications (cfdDNA, AlloMap), and to investigate the utility of this assay in the setting of other solid organ transplants (e.g., lung, kidney, and liver transplantation). Finally, studies that use immune repertoire sequencing results to tailor immunosuppression in individual patients may enable a “personalized medicine” approach to achieving adequate suppression of the alloimmune response, while avoiding the myriad long-term sequelae of over-immunosuppression.

## Supporting Information

S1 Data and Scripts Raw data and Python scripts for the generation of figures. (GZ)

S1 Fig Overall immunosuppression in individuals with and without rejection events. ABS levels (top) are shown for six individuals with and six individuals without moderate-to-severe acute rejection events, as well as for one individual with chronic infection and one individual with amyloidosis. Colors indicate isotype contribution. cfdDNA levels are shown for the same individuals (bottom). Biopsy grades are shown as vertical blue lines (bottom): thin lines indicate mild ACR events (grade 1R), and thick lines indicate moderate-to-severe ACR events (grade ≥ 2R).
(TIFF)

S2 Fig Polyclonal expansion of IgA sequences. IGH molecules are grouped by V and J segment usage and shown as a scatter plot. Every dot represents a V–J combination, with its size indicating the number of molecules featuring that particular V–J combination. Plots are shown for three different time points (onset of post-transplant immunosuppressive therapy [day 1], maximal immunosuppression [day 104], and expansion of IgA [Day 584]). Even distribution of V–J usage suggests polyclonal expansion of IgA sequences.
(TIF)

S1 Table Patient demographics. (PDF)

S1 Text STARD Checklist. (PDF)

## Abbreviations

ABS: activated B cell sequence
ACR: acute cellular rejection
AL: amyloid light chain
cfdDNA: cell-free donor-derived DNA
MMF: mycophenolate mofetil
ROC: receiver operating characteristic
UID: unique identifier
V: variable
