# Supplementary figures and images for: Monitoring Pharmacologically Induced Immunosuppression by Immune Repertoire Sequencing to Detect Acute Allograft Rejection in Heart Transplant Patients: A Proof-of-Concept Diagnostic Accuracy Study
Source: PLoS Med. 2015 Oct 14;12(10):e1001890. doi: 10.1371/journal.pmed.1001890 (PMC4605651; doi:10.1371/journal.pmed.1001890)

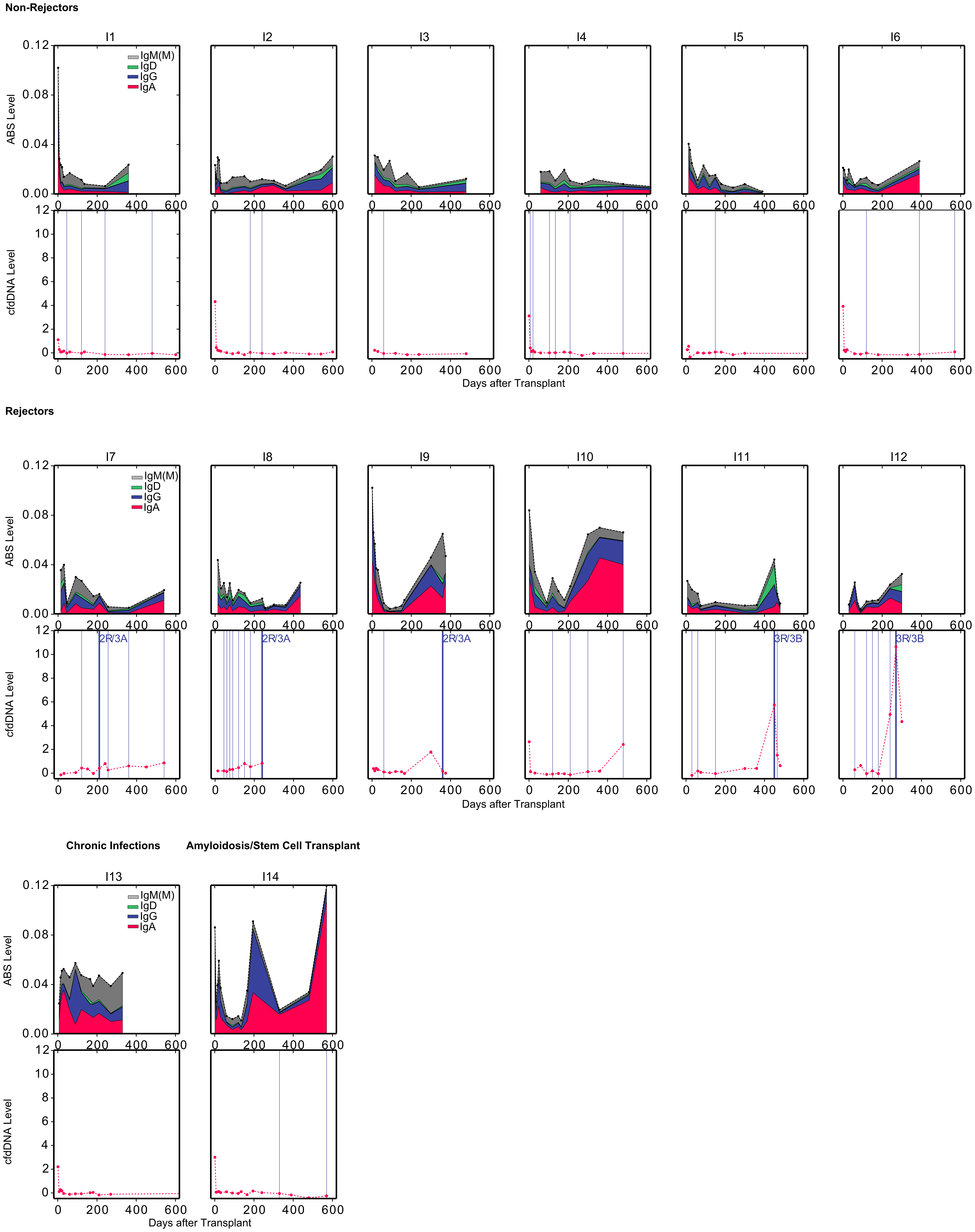

Supplement: S1 Fig — ABS levels (top) are shown for six individuals with and six individuals without moderate-to-severe acute rejection events, as well as for one individual with chronic infection and one individual with amyloidosis. Colors indicate isotype contribution. cfdDNA levels are shown for the same individuals (bottom). Biopsy grades are shown as vertical blue lines (bottom): thin lines indicate mild ACR events (grade 1R), and thick lines indicate moderate-to-severe ACR events (grade ≥ 2R). (TIFF) [file pmed.1001890.s002.tiff]

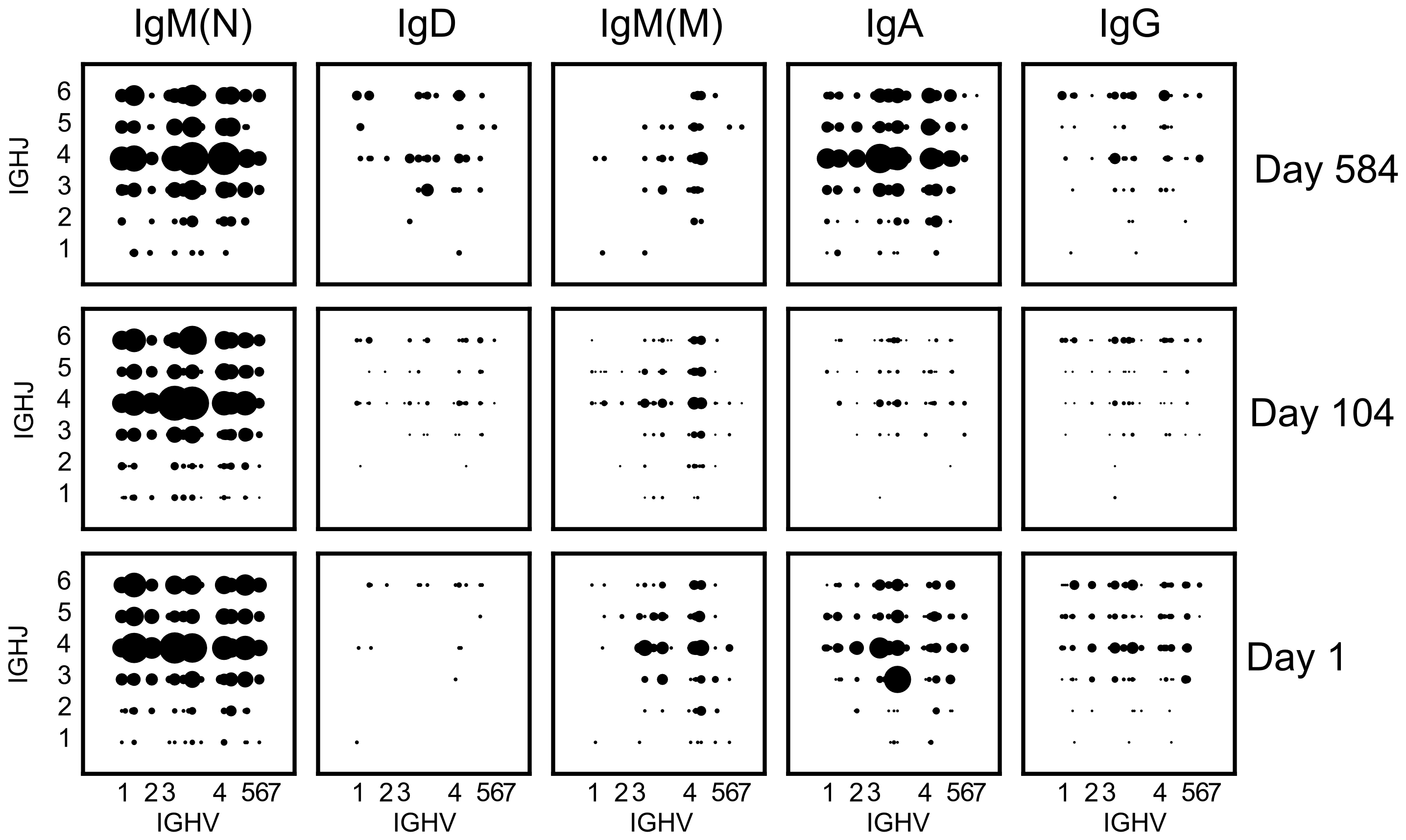

Supplement: S2 Fig — IGH molecules are grouped by V and J segment usage and shown as a scatter plot. Every dot represents a V–J combination, with its size indicating the number of molecules featuring that particular V–J combination. Plots are shown for three different time points (onset of post-transplant immunosuppressive therapy [day 1], maximal immunosuppression [day 104], and expansion of IgA [Day 584]). Even distribution of V–J usage suggests polyclonal expansion of IgA sequences. (TIF) [file pmed.1001890.s003.tif]
