# Supplementary material for: Monitoring Pharmacologically Induced Immunosuppression by Immune Repertoire Sequencing to Detect Acute Allograft Rejection in Heart Transplant Patients: A Proof-of-Concept Diagnostic Accuracy Study
Source: PLoS Med. 2015 Oct 14;12(10):e1001890. doi: 10.1371/journal.pmed.1001890 (PMC4605651; doi:10.1371/journal.pmed.1001890)
Supplement: S1 Table — (PDF) [file pmed.1001890.s004.pdf]

Group	# of Participants	Age (Average ± STD)	Male/Female	Follow-up (Average ± STD)
Non-Rejectors	6	60(±5.5) years	6/0	16±4.5 months
Rejectors	6	36.7(±17) years	1/5	14.5±2.9 months
